# A qualitative study of integrated care from the perspectives of patients with chronic obstructive pulmonary disease and their relatives

**DOI:** 10.1186/1472-6963-14-471

**Published:** 2014-10-02

**Authors:** Pernille Maria Wodskou, Dorte Høst, Nina Skavlan Godtfredsen, Anne Frølich

**Affiliations:** Department of Integrated Care, Bispebjerg University Hospital, Bispebjerg Bakke 23, DK-2400 Copenhagen, NV Denmark; Department of Nursing, Faculty of Health and Technology, Metropolitan University College, Tagensvej 86, DK-2200 Copenhagen, NV Denmark; Department of Pulmonary Medicine, Bispebjerg University Hospital, Bispebjerg Bakke 23, DK-2400 Copenhagen, NV Denmark

**Keywords:** Integrated care, Continuity of care, Delivery of healthcare, Disease management programmes, Patient perspective, Relatives, Chronic obstructive pulmonary disease, Qualitative study

## Abstract

**Background:**

Disease management programmes have been developed for chronic obstructive pulmonary disease (COPD) to facilitate the integration of care across healthcare settings. The purpose of the present study was to examine the experiences of COPD patients and their relatives of integrated care after implementation of a COPD disease management programme.

**Methods:**

Seven focus groups and five individual interviews were held with 34 patients with severe or very severe COPD and two focus groups were held with eight of their relatives. Data were analysed using inductive content analysis.

**Results:**

Four main categories of experiences of integrated care emerged: 1) a flexible system that provides access to appropriate healthcare and social services and furthers patient involvement; 2) the responsibility of health professionals to both take the initiative and follow up; 3) communication and providing information to patients and relatives; 4) coordination and professional cooperation. Most patients were satisfied with their care and raised few criticisms. However, patients with more unstable and severe disease tended to experience more problems.

**Conclusions:**

Participant suggestions for optimizing the integration of healthcare included assigning patients a care coordinator, telehealth solutions for housebound patients and better information technology to support interprofessional cooperation. Further studies are needed to explore these and other possible solutions to problems with integrated care among COPD patients. A future effort in this field should be informed by detailed knowledge of the extent and relative importance of the identified problems. It should also be designed to address variable levels of severity of COPD and relevant comorbidities and to deliver care in ways appropriate to the respective healthcare setting. Future studies should also take health professionals’ views into account so that interventions may be planned in the light of the experiences of all those involved in the treatment of COPD patients.

**Electronic supplementary material:**

The online version of this article (doi:10.1186/1472-6963-14-471) contains supplementary material, which is available to authorized users.

## Background

Chronic obstructive pulmonary disease (COPD) is estimated to affect 9-10% of the adult populations in Europe and North America [1] and 9-14% of the Danish adult population [2, 3], making COPD an important disease to address regionally as well as globally [4]. Periodic exacerbations of COPD symptoms, such as breathlessness and fatigue, often hamper COPD patients’ functional capacity and cause them to become socially isolated, which makes healthcare logistically challenging and not easily accessible. COPD care must therefor be flexible and continuous, which implies that it must span several sectors of the healthcare system [5]. Flexibility and continuity are important aspects of integrated care, and they are key concepts in the planning of health services [6]. Integrated care is needed to meet the health care needs of patients with COPD and other chronic and complex conditions and is being implemented in many countries around the world [6, 7].

The concept of integrated care has been the focus of several studies internationally, but confusion still exists as to the nature and definition of the concept [6, 8–10]. In Denmark, disease management programmes have been developed, inspired by the chronic care model [11], to support the integration of healthcare services and improve the quality and the effectiveness of care. The programmes describe combined multidisciplinary, intersectoral and coordinated efforts for a specific chronic condition. Disease management programmes should take into account patient, clinical and organisational perspectives [12]. The patient perspective is justified not only for its intrinsic value, but also for its positive association with patient safety and clinical effectiveness [13]. However, only a handful of studies have attempted to describe or evaluate disease management programs or the concept of integrated care from the perspectives of patients or relatives. One study among inpatients concluded that patients’ experiences of coherence are intimately linked to their interactions with health professionals, the continuity of the dialogue and ‘everyday life’ in the department during hospital stays. The patient’s perspective differs greatly from the organizational perspective, which emphasises the importance of cooperation and coordination between settings [14]. Thus, patients and organizations seem to stress different aspects of integration.

Qualitative studies among patients with chronic conditions reveal that they experience a number of problems with healthcare services across sectors. These problems relate to access to appropriate and timely care, communication and coordination between settings, relational continuity and patient information, among other issues [15–18]. Patients encountering these problems experience delayed care pathways and delayed adjustment to life with a chronic disease [17]. Furthermore, research suggests that disease severity and/or care structure affect how different groups of patients experience problems related to transitions between care settings, and that these factors should be further explored [16, 19]. Only one study has specifically focused on patients with COPD [18]. Thus, more research is needed to explore the challenges in the healthcare system faced by patients with COPD .

Many relatives take an active role in monitoring and managing COPD patients’ needs. They experience many of the same problems that patients do—such as anxiety, powerlessness and social isolation—yet knowledge about their needs is scarce. Support from health professionals can help relatives cope [5]. However, we need more knowledge in two areas: 1) the experience of integrated care from the perspectives of COPD patients and their relatives and 2) how this experience is shaped by the severity of disease and treatment within different care settings.

Consequently, the aim of the present study was to examine how COPD patients and their relatives experience integrated care across care settings after the implementation of a COPD disease management programme. The results of the study will inform future evaluations of the COPD disease management programme in the region and add to the international literature regarding this important issue.

## Methods

We conducted focus group interviews and individual interviews with COPD patients and their relatives to answer the following research questions:

1. From the perspectives of COPD patients and their relatives, what constitutes integrated care?
2. Where in the care process do COPD patients and their relatives experience a lack of integration?
3. What initiatives do COPD patients and their relatives suggest for optimizing the care process?
4. How do the answers to these questions differ between different groups of COPD patients and their relatives?

The development of the research questions was guided by previous research [14–18] and by the aim of the study.

### Setting

The study took place at Bispebjerg University Hospital in the Capital Region of Denmark, where the COPD disease management programme was implemented three years before the study. The COPD disease management programme guides the stratification of patients into groups and defines where and how patients should be treated. Generally, patients with mild and moderate COPD are monitored by a general practitioner (GP) once a year. Patients with severe COPD are monitored by a GP every three to six months. They are monitored permanently or temporarily in the hospital pulmonary outpatient clinic if they have comorbidities, frequent exacerbations, severe dyspnoea, etc. Patients with very severe COPD are monitored every six months in the outpatient clinic [20].

### Participants

Participants were patients with severe or very severe COPD (forced expiratory volume in one second (FEV_1_) <50% of predicted) [21] living in the City of Copenhagen and their relatives. These patients both see their GP and attend specialist care at the pulmonary outpatient clinic at Bispebjerg University Hospital. Using purposive sampling, we assembled five participant groups: 1) patients attending pulmonary rehabilitation in the municipality, 2) patients attending pulmonary rehabilitation at the hospital, 3) patients never attending pulmonary rehabilitation, 4) patients recently discharged from the hospital following an acute exacerbation of COPD and 5) relatives. Excluded from the study were patients with dementia, mental instability, inability to understand or speak Danish or patients who were unable to participate in a focus group due to their physical condition. The latter exclusion criterion was waived for patients recently discharged from the hospital, who were interviewed individually in their homes.

A project nurse screened health records of possible participants attending the outpatient clinic. If they met the inclusion criteria, they were contacted by telephone or in person in the outpatient clinic and informed about the study. To recruit relatives, we contacted patients with severe or very severe COPD and asked if they would allow us to contact a relative. Relatives were then contacted in the same manner as patients. Patients and relatives who agreed to participate were mailed a written invitation and information sheet. In preparation for the study, a pilot focus group interview was conducted with patients who met the inclusion criteria. Pilot participants were randomly selected at the outpatient clinic.

Forty-four of 64 COPD patients and eight of 16 relatives approached about the study accepted the invitation to participate. Twenty patients declined, seven patients declined on behalf of their relatives and one relative declined. Eight patients cancelled participation due to illness, and two patients did not show up for the interviews. A total of 34 patients and eight relatives eventually participated in the study. The characteristics of participants, groups and non-participants are shown in Tables 1 and 2.

**Table 1:** **Participant and group characteristics (n = 42)**

Patients, n	34
Age in years: median (range)	72 (48–87)
FEV_1_ (% of predicted): median (range)	35.0 (18.5-49)
Gender: male/female, n	15/19
Last admission due to COPD: < 1 year ago/> 1 year ago/ never, n	9/14/11
Pulmonary rehabilitation: municipality/ hospital/ municipality and hospital/never, n	8/12/2/14
**Relatives, n**	**8**
Gender: male/female, n	3/5
Relation: husband/wife/daughter, n	3/3/2
Patients’ age in years: median (range)	68.5 (56–79)
Patients’ FEV_1_ (% of predicted): median (range)	26 (18–49)
**Number of participants in groups**	
Pilot (one group), n	6
Pulmonary rehabilitation in the municipality (two groups: a + b), n	4 + 5
Pulmonary rehabilitation at the hospital (two groups: a + b), n	4 + 5
No pulmonary rehabilitation (two groups: a + b), n	2 + 3
Recently discharged (individual interviews), n	5
Relatives (two groups: a + b), n	4 + 4

**Table 2:** **Characteristics of non-participants**

Non-participants, n		38
Age in years*: median (range)		74 (42–90)
FEV_1_ (% of predicted)*: median (range)		38.5 (17–48)
Gender: male/female*, n		14/16
Invited for group:	Pilot, n	2
	Pulmonary rehabilitation in the municipality, n	7
	Pulmonary rehabilitation at the hospital, n	6
	No pulmonary rehabilitation, n	10
	Recently discharged, n	5
	Relatives, n	8
Reason for non-participation:	No reason, n	13
	Feeling too unwell, n	10
	Dates did not match, n	5
	Patients declining on behalf of relatives, n	7
	Miscellaneous (miscommunication, nervous to speak in front of strangers, recently moved to nursing home), n	3

*Data for patients were available (n = 30).

### Data collection

Focus group interviews were chosen because they are suitable for achieving an understanding of the perspectives and opinions of a particular group [22] and for generating impressions of and identifying problems with the services and programmes being investigated [23]. A semi-structured interview guide was developed, based on the study aim and research questions [22–24]. The interview guide was revised after feedback from other researchers and in light of the results of the pilot focus group (for a translated version of the interview guide, please see the Additional file 1).

We aimed to recruit seven or eight participants for each focus group, hoping for five or six participants. We judged that small focus groups would be appropriate because of participants’ physical limitations and the relative complexity of the subject [23]. To increase attendance, we offered participants free transportation by taxi to and from interviews. We aimed to generate homogeneous groups of participants who did not know one another by using segmented focus groups based on participant types (patients attending pulmonary rehabilitation, relatives, etc.) to stimulate discussion. We also sought to include men and women of varying ages in all focus groups to include diverse experiences and opinions [23]. Focus group interviews were held at the hospital in a building separate from the clinical departments, which was unfamiliar to participants. The interviews lasted 90–120 minutes, including a ten-minute break, and were audio recorded and transcribed verbatim.

A total of nine focus group interviews, including the pilot interview, and five individual interviews were held. As the series of interviews progressed, data saturation occurred.

### Analysis

All 14 transcribed interviews were coded and categorised inductively using qualitative content analysis [25–27], primarily inspired by Graneheim and Lundman [26], and supported by the qualitative data analysis software NVivo 10. We focused primarily on the manifest content of data [26, 27]. Transcribed interviews were first read in their entirety to obtain a sense of whole [25, 26]. The text was then divided into units of meaning that were condensed and grouped together to create categories and sub-categories [26]. During this process, the content of sub-categories and categories was continuously compared to data in other emerging categories to ensure that the data in categories belonged together and not to other categories [25]. The analysis resulted in four categories with a total of 12 sub-categories.

The categories that emerged shed light on the perspectives of COPD patients and their relatives on integrated care: 1) a flexible system; 2) initiative and follow up; 3) communication and information; and 4) coordination and cooperation. The wording of category titles was derived primarily from organizational terminology related to the concept of integrated care. Comparative analysis using NVivo 10 software identified possible patterns in problems related to integrated care among the five different types of participants.

Coding and categorisation of content was discussed by the analyst and another researcher to provide validation of the process and findings [26]. A third researcher not involved with the study also recoded 72 selected units of meaning into categories; coding-recoding agreement was 92%.

The researchers primarily involved in the data collection and the analysis were not employed by the Department of Pulmonary Medicine nor had they had any contact with the patients in the Department of Pulmonary Medicine.

Reporting of the study adheres to the RATS guidelines for reporting qualitative studies (http://www.biomedcentral.com/authors/rats).

### Ethical considerations

All participants gave informed written consent and were guaranteed anonymity and confidentially. The Danish Data Protection Agency approved the collection and management of data in the study. Ethical review by a health research ethics committee is not required for qualitative studies in Denmark [28].

## Results

The results of the analysis in the form of **categories** and their respective *sub-categories* are described in the following section. Most patients were satisfied with their care and voiced few criticisms. However, to identify areas needing improvement, focus inevitably turned to the problems. As a result, the description of the care process likely became more negative than what most participants actually experienced. The areas of the care process with which patients were satisfied were generally the same ones in which other patients experienced problems, e.g., many patients experienced continuity of care in the pulmonary outpatient clinic, but some did not. The comparative analysis between the segmented focus groups revealed only few differences. These differences are described under the relevant categories. Participants’ suggestions for optimizing the care process are described together because some suggestions covered problems that cut across categories. Quotations provide key examples of participants’ experiences.

### A flexible system

This category describes a patient-centred approach to the concept of integrated care, in which the system adapts to patients’ needs and not vice versa. According to patients and their relatives, a flexible system is one in which patients and relatives have access to staff and services when they need them, patients are involved to the extent they desire in decisions regarding their care, and appropriate healthcare and social services are available and able to accommodate individual patients.

#### Access

The term access refers to the availability of health professionals and services when needed. Access provides a sense of security and is particularly important for participants when symptoms worsen and patients and relatives are unsure about what to do. Some participants had difficulty accessing their GP when symptoms worsened because many GPs are only open for emergency contacts from 8 to 9 am.

*“And if I suddenly wake up and feel very poor, for example at 10 am, then it’s over. I try calling (the GP’s office) to get an appointment. But it is simply impossible: “We can’t today, he is busy.”.”* (Female patient, focus group with patients participating in hospital-based pulmonary rehabilitation).

In addition, many patients reported that their GP’s office was located in a building without elevators. Absence of elevators limits many COPD patients’ access to their GPs for both emergency and routine consultations. Some participants had GPs that made house calls instead.

Participants reported that they had no access to the pulmonary outpatient clinic outside of scheduled appointments, but they expressed a wish for easier access, e.g., over the telephone or by computer. Furthermore, patients waited months after referral to see a specialist, and many participants felt that the six-month interval between appointments was too long and that the length of this period affected their symptoms and caused uncertainty. Some patients experienced being discharged from the outpatient clinic without an explanation and against their will. These patients felt unsafe and abandoned, but they found comfort in knowing that they could call an ambulance if they needed to and that a hospitalization would make it possible for them to return to the outpatient clinic.

Several participants found it difficult to become admitted when they suffered a COPD exacerbation and they felt hospitalization was needed. In addition, many patients who were hospitalized experienced being discharged too soon, which they believed led to rapid readmissions.

#### Patient involvement

Patient involvement was a lively topic of discussion in the focus groups because participant views differed greatly. Some patients experienced always being involved, some felt that they were only involved if they were “impertinent” or “big-mouthed”, and some felt that they were never involved. It was generally easier for patients to be involved in decisions regarding medication and timing of appointments, even if it was not always possible for the staff to accommodate their wishes. On the other hand, it was difficult to be involved in decisions regarding access, e.g., discharge from the outpatient clinic and the pulmonary ward.

#### Appropriate healthcare and social services

According to participants, patients need appropriate healthcare and social services that align with their perceived needs to be able to care for themselves and perhaps avoid hospitalization. The analysis showed that what amounts to appropriate care is, indeed, highly individual because different patients and families have different resources to cope with their problems and therefore require different care or services. Care and services were perceived as being limited in three ways. First, some offers of care and services did not match patients’ needs; for example, many patients said the rehabilitation course ended just as the training began to have an effect. Second, participants experienced being denied appropriate care and services due to bureaucratic rules, e.g. eligibility for services like transportation or home oxygen therapy could depend on a marginal difference in lung function or on age and retirement status. Third, some needed services did not exist. The latter was primarily expressed by relatives, who felt a need for consultations with a physician or psychologist themselves and for support from peer groups to cope with feelings of insecurity, distress and loneliness.

### Initiative and follow up

Initiative and follow up were deemed to be critical to the integrated nature of the care and the services offered. Participants wanted and expected health professionals to take responsibility for initiating relevant actions and to subsequently follow up on these actions.

#### Initiative

Participants expected health professionals to consistently be well informed about COPD, consider appropriate diagnostic investigation and treatment, and take the initiative to talk to patients about options for rehabilitation, tests, home help, etc. Some patients wanted to be contacted with relevant information, e.g. about pulmonary rehabilitation, influenza vaccination and laboratory results. This request was expressed predominantly by patients who did not know how to use a computer and by some patients who had not attended pulmonary rehabilitation and were hesitant to do so. Participants needed health professionals to take the initiative because patients lacked knowledge about relevant treatment and care options and disliked asking for anything.

#### Follow up

Participants said that health professionals need to follow up on actions that had been implemented. Following up involved evaluating the problem that was addressed and the action taken and planning ahead, including initiating new actions as needed. In this regard, there seems to be a circular relationship between the sub-categories of initiative and follow up. Participants did not consider sufficient the scheduled annual/biannual follow ups at the GP or outpatient clinic because COPD patients’ problems continuously changed and subsequent treatments and services needed to change accordingly. With respect to hospital discharges, some patients requested that the GP, his or her secretary or a community nurse telephone the patient and follow up on the admission and plan new actions to facilitate the transition from hospital to home, as exemplified by this patient’s comment:

*“When the health centre (the community nurses headquarter) is told that I have come home, then I would like them to contact me, because I do need them to come.”* (Individual interview, female patient recently discharged from hospital)

When there was no follow up, the care process stopped, which affected patients’ physical and mental well-being as well as the relationship between patients and health professionals. Examples of a lack of follow up included patients not having suggested tests because the order was never forwarded to the lab, home help suddenly ending without a reassessment of the patient’s needs, and a newly diagnosed patient who went untreated for 18 months which caused a rapid decline in lung function.

### Communication and information

This category relates to personal interactions between patients and their relatives and health professionals. The analysis showed that both the quality of interactions and the information provided by health professionals were affected by the atmosphere of the interaction. Participants requested empathy and cooperation, competent professionals, sufficient time and an appropriate physical environment.

#### Empathy and cooperation

When patients were in contact with health professionals, they felt that it was important for their well-being that they were greeted with a smile, considered equals, and met with helpfulness and understanding. These factors were considered conducive to joint decision-making about treatment. A few patients had experienced being treated in a derogatory or even rude way by nurses and physicians in the outpatient clinic, and they accordingly felt humiliated and angry and that they imposed a burden on healthcare professionals. Questions about smoking sometimes felt humiliating to patients, who requested that health professionals show more tact when addressing the issue.

#### Information

Patients differed in terms of the amount and type of information they wanted to receive; some participants wanted to know everything about disease severity and prognosis and others wanted as little information as possible. However, most patients were satisfied with the level of information in general and felt well informed, particularly when written information was followed up verbally. Many participants thought that patients are responsible for asking questions if they need more information. However, some patients did not feel well informed and for various reasons did not ask questions. A problem voiced by several respondents was that health professionals spoke as experts, i.e., they used medical terminology that patients did not understand, but patients did not ask questions because they did not want to interrupt or did not share an empathic connection with the health professional. In addition, different health professionals sometimes had varying opinions about a patient’s care, and the conflicting information caused confusion and uncertainty for patients. Participants wished that health professionals would give high priority to information, because they perceived it as essential for developing a sense of security and confidence.

Responses from patients and their relatives about the information needs of relatives differed greatly. Patients thought that relatives needed information about what it is like to have COPD. Conversely, relatives wanted information about how to manage everyday life. Many relatives experienced unmet information needs.

#### Competent professionals

Participants expected competence from professionals at all levels from home helpers to physicians, and many participants were happy with the level of competence they experienced from healthcare professionals. Most participants preferred contact with specialists over contact with GPs because of the specialists’ expert knowledge and because many patients had experiences of GPs neither caring nor having sufficient knowledge about COPD: *“I don’t see my GP that often because they don’t know so much about it (COPD)”.* (Male patient, pilot focus group).

#### Setting

Setting includes time and the physical environment. Many participants found that inadequate time to properly address issues raised by patients was a general problem in both the primary and secondary sectors. Problems related to the physical environment pertained largely to the pulmonology ward in the form of too many patients, not enough staff and poor toilet and shower facilities. As a consequence, some patients felt that the nursing staff failed to provide appropriate care.

### Coordination and cooperation

This category concerns organisational and relational aspects of integrated care. It contains the sub-categories of coordination, relational continuity and professional cooperation.

#### Coordination

Participants mostly described experiences of coordination that took place between settings when something went wrong. However, several patients described the care process as ‘coherent’ or ‘good’ when they were automatically sent to different parts of the care system and received appropriate healthcare and services. The fragmented nature of the system became clear when patients or their relatives needed to contact several different authorities to ensure coordination of care, when mistakes happened during transitions between settings, e.g., home help not showing up after hospital discharge, and when professionals from different organizations disagreed on appropriate care and services. In one case, health professionals in the secondary sector recommended specific services delivered by the municipality, but the municipality turned down the application

*“It does not matter whether you have eight psychiatrists and hospitals that recommend the one and the other. The municipality has employed some people to just say no. What should we do? They say no anyway”.* (Male relative)

#### Relational continuity

Most participants appreciated relational continuity, which they described as ‘knowing each other’. In particular, patients with very severe illness who had been admitted to the pulmonology ward and had many contacts with the staff in the outpatient clinic experienced becoming a familiar face in the clinic and knew several physicians and nurses. Relational continuity had a positive impact on the patient’s sense of security, confidence and understanding and on the experienced quality of healthcare; patients perceived the system as more flexible and health professionals as taking more responsibility and initiative and following up. Many patients experienced relational continuity with their GP and in the outpatient clinic and, to a lesser extent, in the inpatient pulmonary ward and in the municipality. In the absence of relational continuity, the main problem was that patients repeatedly had to answer the same questions and explain the same things because, for instance, a new physician did not have time to thoroughly read the often very thick patient file. This experience felt annoying and frustrating to patients, and some patients declined care or services as a result.

#### Professional cooperation

Effective professional communication across organizations was deemed important by participants. Some patients had the impression that the flow of information ran smoothly, while others experienced no form of cooperation. Many patients and relatives contributed to professional cooperation by updating medication lists and providing them for various health professionals. Participants reported that non-communication resulted in problems related to follow up and to assigning healthcare and social services that required information from other professionals. As a result, patients with very severe disease were most affected by insufficient professional cooperation due to their more comprehensive use of services in several parts of the system.

### Suggestions for optimization of the care process

Patients recently discharged from hospital and their relatives advocated for the introduction of a coordinator as a possible solution to problems related to access, follow up and coordination. The descriptions of the coordinator’s role depended on the concrete problems participants were experiencing. Examples of a coordinator included: a nurse with expert knowledge about COPD to guide patients and relatives about how to cope with exacerbating symptoms; a health professional or social worker who would contact the patient every three months or so to see how things were going in the family and address problems before they escalated; a social worker who could provide financial assistance and help the patient receive aid and devices to which he or she was entitled; and a health professional or a social worker in the municipality who was the only professional the patient or relative needed to contact, who would then request and coordinate healthcare and social services across sectors. When asked, patients who had never participated in pulmonary rehabilitation felt that a coordinator was unnecessary.

To achieve a more flexible system, self-reliant patients requested more self-monitoring in relation to scheduled follow ups and housebound patients requested telehealth solutions. Participants also advocated for easier access to the outpatient clinic.

Participants suggested bringing specialist care into patients’ homes by educating and equipping GPs, community nurses and home helpers to care for patients at home with stable symptoms and, during periods of symptom instability, to prevent or delay hospitalization.

Implementation of a common IT solution to which all relevant health professionals and social workers had access was the most frequently mentioned suggestion for facilitating cooperation between professionals.

## Discussion

The study showed that COPD patients and their relatives view the care process as integrated when:

- The system is flexible, provides access to staff, appropriate healthcare and social services that match patients’ and relatives’ needs, and involves the patient.
- Health professionals take the initiative to suggest and implement relevant actions and subsequently follow up.
- Communication and information involves empathy and cooperation, competent health professionals, adequate time and a suitable physical environment.
- The care process is coordinated within and between care settings in a manner that facilitates relational continuity and professional communication.

Participants experienced both care integration and the lack thereof throughout the care process. In all categories and sub-categories, some participants reported that care integration was present and others reported that it was absent. Comparative analysis showed only two systematic differences. First, patients recently discharged from the hospital and relatives were more prone than other patients to experience more problems and to advocate for a care coordinator to help them coordinate the care process. Second, relatives expressed needs for information and support from health professional and peers that were not acknowledged by patients in this study.

### Comparisons with other studies

The results largely agree with previous studies about patients’ experiences with chronic illness care [15–18], as will be described thematically.

### Access

The sub-category of ‘access’ shares many similarities with the theme ‘Getting in’ from the research by Preston et al. [17]. Problems with access seem not to be unique to patients with COPD, but some differences between our and previous research were found. Participants described most health professionals in our study as helpful and trying to accommodate patients’ wishes, e.g., in relation to the timing of appointments. In other studies, patients found staff to be unwilling to accommodate their wishes concerning the timing of appointments [16], and they identified the attitudes of the reception staff and the patient/doctor relationship as barriers to access [17]. The attitudes of health professionals do not explain the problems related to access in our study, whereas the patient-doctor relationship could affect patients’ experiences of access. Thus, both our study and others [15, 17] found that relational continuity affects patients’ involvement and their experience of flexibility. Problems with access in the hospital sector can also be examined in light of some of the other problems that appeared in our study. Many patients felt that too little time was allocated to each patient in the outpatient clinic, it was too difficult to be admitted, nurses in the pulmonology ward were too busy and hospitalized patients were discharged too soon. In this light, the problems of access and problems related to the setting could signify that the supply of resources in the form of hospital beds and health professionals did not match the demand, particularly in relation to the number of COPD patients with complex care needs. From the perspectives of COPD patients and their relatives, integrated care thus seems to require more resources than are currently available, for example, in the form of easier access to the outpatient clinic as several participants suggested*.*

### Follow up

Follow up was an important aspect of integrated care for all participants in our study and for patients in other studies [16]. Participants did not reflect on the particular challenges of coordination across different healthcare settings to achieve successful follow up. Some participants described the concept of coherence as an automatic process in which they were sent from one setting to another to receive appropriate care and services. This form of coherence requires that organizational structures are in place to support the care process and that health professionals in different parts of the system know about healthcare options for patients in general and take the initiative to refer patients to other healthcare settings. Thus, the concept of integrated care comprises both an organizational aspect and a professional aspect.

### Communication and information

Participants in our study and others stressed the importance of health professionals meeting patients with empathy and as equals, and that it is important that adequate time be set aside for patients to be properly informed [15–18]. Appropriate information provides patients with reassurance, builds confidence and a sense of control [17] and helps them navigate the system [18]. Like Berendsen et al. [15], we found that the amount and type of information patients want varies greatly. In addition, problems related to information found in this study are consistent with those found in other studies, including conflicting information [15, 17] and the use of medical language that patients do not understand [15].

### Relational continuity

Relational continuity was valued by most participants in our study and in others [16, 17]. Among patients in our study, relational continuity meant that the patient and health professional knew each other and had formed a relationship. As Cowie et al. [16] expressed it, they share a personal and clinical history.

Many participants preferred being in contact with the specialist rather than the GP, and some patients experienced their GP as uninterested in or unknowledgeable about COPD. This finding contrasts with other studies in which patients prefer professional contact with their GP, as compared to other healthcare professionals, because of the higher degree of relational continuity [15, 17]. The difference is quite interesting, and there are several possible explanations for this finding. First, many patients in our study experienced relational continuity with their specialists, although patients in other studies described specialist contacts as impersonal. Second, patients in other studies suffered from a variety of chronic diseases, and the finding of relational continuity with specialists may be specific for patients with COPD. Third, all patients in our study had severe or very severe COPD, and thus required specialist care. Cowie et al. [16] found that patients receiving specialist-led shared care navigated secondary care more easily than did patients receiving GP-led shared care. Thus, familiarity with specialist care could also be a factor.

### Relatives

Our study confirmed the findings of other studies that relatives of COPD patients need support from healthcare professionals [5]. However, our study also documents the lack of support relatives receive in the form of, for instance, information about coping with daily life or support from a psychologist or a peer group. Research has shown that failure to support and relieve relatives in caregiving roles can result in burnout [5].

### Suggestions from patients and relatives to optimize the care process

Suggestions to optimize the care process included assigning a care coordinator to individual patients. In the literature, the terms care coordinator and case manager have often been used interchangeably, and the definitions of roles depend both on the setting and the profession of the coordinator, e.g., nurse, physician or social worker [10]. Nurse care coordinators provide, among other things, a single point of access, timely needs assessment, symptom management, multidisciplinary coordination and transition planning [29], as participants also requested in our study. However, participants had very different ideas about what knowledge and responsibilities a care coordinator should have, and further research about the role of the care coordinator is needed. Participants also proposed that the care process could be optimised by better information technology to support interprofessional cooperation, which is also identified in the literature as an essential element in building integrated care [8]. They also suggested that telehealth solutions could improve access. Telehealth solutions have a positive impact on patients’ quality of life and healthcare usage [30].

### What this study adds

In general, our study findings are consistent with those of previous research, although there are a few exceptions. Significant findings of our study relate to the views of COPD patients and their relatives and the differences between them, the relationship of patients’ views to their position on the care pathway and participants’ focus on opportunities for optimizing the care process.

### Strengths and limitations of the study

Focus groups worked well to meet the purpose of the study. Both agreements and disagreements were freely shared in the groups. Patients recently discharged from the hospital could not attend focus groups and were interviewed individually. They provided insight into the experiences of the sickest patients that would otherwise have been lacking.

The analysis supported the development of inductively inferred categories. During the analysis, emerging categories were discussed and agreed upon by the analyst and a co-researcher, which strengthens the validity of the results.

During interviews, problems and ideas mentioned in earlier interviews were sometimes brought up by the moderator when deemed appropriate to the discussion. It is possible that participants in earlier focus groups would have had opinions about some topics, such as care coordinators, that arose in later groups. This both narrows the strength of the comparative analysis and strengthens the findings regarding the other research questions because views and ideas expressed earlier in the study were investigated further.

Only half (42/80) of eligible participants eventually participated. Patients who never attended pulmonary rehabilitation were the most difficult to recruit; only five of 15 eligible patients participated. The patients who did not participate most frequently said that they were feeling too unwell to participate, which indicates that patients with fewer symptoms and problems chose to participate. Given the likelihood that patients with more unstable and severe disease experienced more problems, it is likely that our findings reflect a more positive aggregate view of the care process than would have been the case had we succeeded in recruiting a more representative sample. The opposite can possibly be said for the relatives. The primary reason given for non-participation among relatives was that patients had declined on their behalf because they did not want to impose on them. It is likely that participants who were relatives heard about the study because they were more involved with their family members’ care. This may indicate that they were relatives of patients with more severe illness, which was also suggested by a low median FEV_1_ (% of predicted) of patients in this group (Table 1).

The results are based on the views and experiences of 34 COPD patients and eight relatives. They were recruited from the same hospital and municipality in Denmark. They share geography, healthcare and a social system. However, the similarity of our findings to those of other European studies [14–17] involving chronically ill patients suggests that problems identified by participants are not unique to either geography or disease.

## Conclusions

The COPD disease management programme involved integrated care. To patients and relatives, integrated care was provided within the framework of a flexible system granting patients access to appropriate healthcare and social services and involving patients; within this system, health professionals take initiative and are responsible for following up, communication and providing information to patients and relatives, and coordination and professional cooperation. Based on this and previous studies [14–18], we acquired an understanding of integrated care from the perspectives of chronically ill patients. What is needed now regarding the patient perspective is further exploration of possible solutions to the problems described, quantitative assessment of the scale and distribution of these problems and possible solutions in different diagnostic and care settings. In addition, it would be useful to investigate the experiences and views of health professionals and to plan interventions to overcome difficulties experienced by patients, relatives and health professionals.

## Electronic supplementary material

Additional file 1: **Interview guide.** The translated version of the interview guide. (PDF 229 KB)

## Abbreviations

COPD: Chronic obstructive pulmonary disease
GP: General practitioner
FEV_1_: Forced expiratory volume in the first second.
