# Supplementary material for: A qualitative study of integrated care from the perspectives of patients with chronic obstructive pulmonary disease and their relatives
Source: BMC Health Serv Res. 2014 Oct 2;14:471. doi: 10.1186/1472-6963-14-471 (PMC4283082; doi:10.1186/1472-6963-14-471)
Supplement: Supplementary file 1 — Additional file 1: Interview guide. The translated version of the interview guide. (PDF 229 KB) [file 12913_2013_3542_MOESM1_ESM.pdf]

Additional file 1

Interview guide

Introduction

- Welcome and thank you for participating in this study. Presentation of moderators: from the Department of Integrated Care at Bispebjerg Hospital, originally a nurse and physiotherapist, but we do not work within the pulmonary field and cannot answer questions about COPD, treatment, etc.
- Expected duration of interview 1-2 hours, incl. break.
- The reason we are here today is that in recent years, Bispebjerg Hospital, the municipality of Copenhagen and the GPs have been working on something called disease management programmes. These programmes involve cooperation, coordination and communication between hospitals and GPs, distribution of responsibilities and tasks, and collaboration with patients and relatives.
- The purpose of the interview today is to obtain knowledge about how COPD patients / relatives of COPD patients experience integration in the care process that involves GPs, the hospital and the municipality, and to find out what can be done better.
- The interview is recorded digitally, and will be analysed alongside some other focus groups we are conducting. It all ends up with us giving recommendations to Bispebjerg Hospital, the municipality and the GPs on how to improve the care process for patients with COPD.
- PW's role in the interview is to ask questions, follow up on what you say and make sure that we have time to talk about all the things we want to know about. Sometimes in focus groups, the conversation drifts away from the focus of the interview. If that happens, I will interrupt and lead the conversation back on track. DH will mostly listen and take notes, but she will probably also say something or ask a question during the interview.
- The interview is anonymous. When we leave after the interview, nobody outside this room will know who was here and who said what.
- In order to get as much information as possible from this interview, it is important:
    - That both positive and negative experiences are told
    - That everybody participates and says something
    - That you do not interrupt each other
    - Remember: no right or wrong answers.
    - Relax and talk freely
- Any questions?

Interview

1. First, we start with a presentation round. Start by briefly saying your name, how long you have had COPD and where you usually go to receive treatment.

2. Now, I will ask you to take a few minutes and think back on your COPD care process – from the time you first experienced symptoms until now. And think of all the contacts you have had in this regard. My question is: what is care integration in the COPD care process?

3. Who will start by telling what he /she thinks about integration in the COPD care process?

4. Now we've talked about X, X and X that means something in relation to integration. What about:
   a. Cooperation and communication between
      i. GP and outpatient clinic?
      ii. Rehabilitation course, GP and clinic?
      iii. Hospital and GP in connection with admission / discharge?
      iv. Hospital and home care in connection with admission / discharge?
5. Overall, do you feel that it is the same doctors, nurses, physiotherapists, etc. that you have contact with? Or is it mostly someone different?

6. What about your influence on your own care process? Do you feel that you may influence decisions?

7. Do you experience that the healthcare system takes into account your individual wishes and needs? Or is it you as the patient who adapts to the system?

8. Have your relatives / you as relatives been involved in the care process?

9. Do you receive the information that you need? (disease management, practical things)

10. Now, I'll change the subject slightly. I would like you to tell me what you think it takes to avoid some of the issues we have talked about today.
    a. What could professionals do?
    b. What could patients do?
    c. What are the three most important things to do?

11. What problems do you see with respect to making the COPD care process better?

12. How we are soon coming to an end. Dorte has written some notes along the way, so that we can better remember the most important things that were talked about today. She will just read them to us and then we check with you that you agree. (DH gives summary of the main points).

13. Is that correctly understood? Did we miss anything?

Closing remark

Thank you so much for participating today. You are welcome to contact us if there is anything in relation to the interview that you would like to talk about. Now we are in the process of interviewing and analysing, and so we hopefully end up with some good recommendations for healthcare and what it takes to make things better.

Probes:

- Can you say something about what makes X important for integration?
- What does X mean to you?
- Can you give some examples?
- What are the implications of X?
- How serious are the consequences for the care process?
- How often do you experience problems in relation to X?
- What do you think when you experience problems?
- What do you do when you experience problems?
